# Emerging trends in DNA and RNA methylation modifications in type 2 diabetes mellitus: a bibliometric and visual analysis from 1992 to 2022

**DOI:** 10.3389/fendo.2023.1145067

**Published:** 2023-05-02

**Authors:** Cai Jiang, Yue Hu, Sinuo Wang, Cong Chen

**Affiliations:** ^1^College of Rehabilitation Medicine, Fujian University of Traditional Chinese Medicine, Fuzhou, China; ^2^Rehabilitation Industry Institute, Fujian University of Traditional Chinese Medicine, Fuzhou, China; ^3^National-Local Joint Engineering Research Center of Rehabilitation Medicine Technology, Fujian University of Traditional Chinese Medicine, Fuzhou, China

**Keywords:** type 2 diabetes mellitus, DNA methylation, RNA methylation, CiteSpace, bibliometrics

## Abstract

**Background:**

Type 2 diabetes mellitus (T2DM) is a pathological metabolic disorder induced by the interaction of genetic and environmental factors. Epigenetic modifications, especially DNA and RNA methylation, might be the bridge between hereditary and environmental factors. This study aimed to comprehensively analyze the status and prospective trends of the association between T2DM and DNA/RNA methylation modifications by using bibliometric software.

**Methods:**

All the publications in the Web of Science database for the research of T2DM with DNA and RNA methylation modifications were obtained from the earliest mention to December 2022. CiteSpace software was used to analyze countries, institutions, journals/cited-references, authors/cited-authors, and keywords. Results of the comprehensive visualization and bibliometric analysis were displayed relative to the research hotspots and knowledge structure.

**Results:**

A total of 1,233 publications related to DNA and RNA methylation modifications and T2DM were collected. The number of publications per year and the overall trend consistently and significantly increased during the investigation period. Based on the highest publication counts, the most influential country was the USA, while Lund University was the most productive institution. DIABETES was considered the most popular journal. The most frequent keywords identified in the field of methylation and T2DM were mainly involved in developmental origin, insulin resistance, and metabolism. The study suggested that the study of methylation modifications had an increasingly significant role in understanding the progression of T2DM.

**Conclusion:**

CiteSpace visualization software was utilized to investigate the status and trends of DNA and RNA methylation modifications in the pathology of T2DM over the past 30 years. Findings from the study provide a guiding perspective for researchers regarding future research directions in this field.

## Introduction

Type 2 diabetes mellitus (T2DM) is a metabolic disorder with a rising global incidence. T2DM mainly manifests in itself pathological changes in insulin secretion and sensitivity resulting from the interaction of genetic and environmental effects (1). The epigenetic phenotype induced by the environment usually depends on epigenetic regulation. Recent accumulative evidence has proven the significant roles of epigenetic aberrations in the initiation and progression of T2DM (2, 3). Further insights into the underlying effects of the epigenetic modifications on the occurrence and improvement of T2DM might facilitate a better understanding of the pathophysiological mechanisms of T2DM. Moreover, the reversibility of epigenetic modifications may hold promise for new ideas and therapeutic approaches in T2DM.

Epigenetic modifications can trigger changes in heritable phenotypes primarily by affecting chromatin modification and do not involve alterations of the nucleotide sequence (4). Unlike genetic abnormalities that are irreversible, epigenetic adaptation and its regulating factors have a regulatory effect on gene activity and expression in response to environmental change (5). This means that the environment, especially early T2DM, can alter an organism’s phenotype (6). As an important epigenetic modification, methylation modification is a biochemical process in numerous reactions that is closely associated with gene regulation and includes DNA and RNA methylation (7). DNA methylation regulates gene expression predominantly by maintaining genomic stability, cell differentiation, and embryonic development (8). In this regard, mounting evidence indicates that DNA methylation is proven to be one important mechanism for maintenance of cellular metabolism such as β-cell dysfunction, insulin resistance, and other conditions, and its abnormality would ultimately trigger the pathogenesis of T2DM (9). Besides, RNA methylation mainly regulates eukaryotic gene expression via post-transcriptional processes (10). Numerous studies have indicated that the progression of T2DM is closely associated with dynamic methylation modifications but using methylation modifications to reverse T2DM remains an urgent problem. Consequently, it is necessary to present researchers with the status, research trends, and frontiers in the field to facilitate future research.

Bibliometrics is a popular and rigorous tool to explore and analyze scientific research output and trends (11). This method summarizes the number of publications in specific research fields and allows researchers to obtain relevant information, including individuals, institutions, countries, Journal Impact Factor, and citations. As a systematic analytical technology, bibliometrics may provide future researchers with valuable information to help them track hotspots and trends and guide clinical policies (12).

To date, no bibliometric study on the association between T2DM and DNA/RNA methylation modifications has been performed. Therefore, a comprehensive visualization and bibliometric analysis of the DNA/RNA methylation in T2DM was conducted in this study to reveal hotspots and prospective trends in apparent modification.

## Materials and methods

### Data source

The Web of Science (WOS) has the most comprehensive information resource with the most detailed citation database. To ensure the comprehensiveness and quality of the data, both the Science Citation Index Expanded (SCIE) and the Social Science Citation Index (SSCI) databases are utilized on the WOS.

### Data preparation

**Figure 1:**
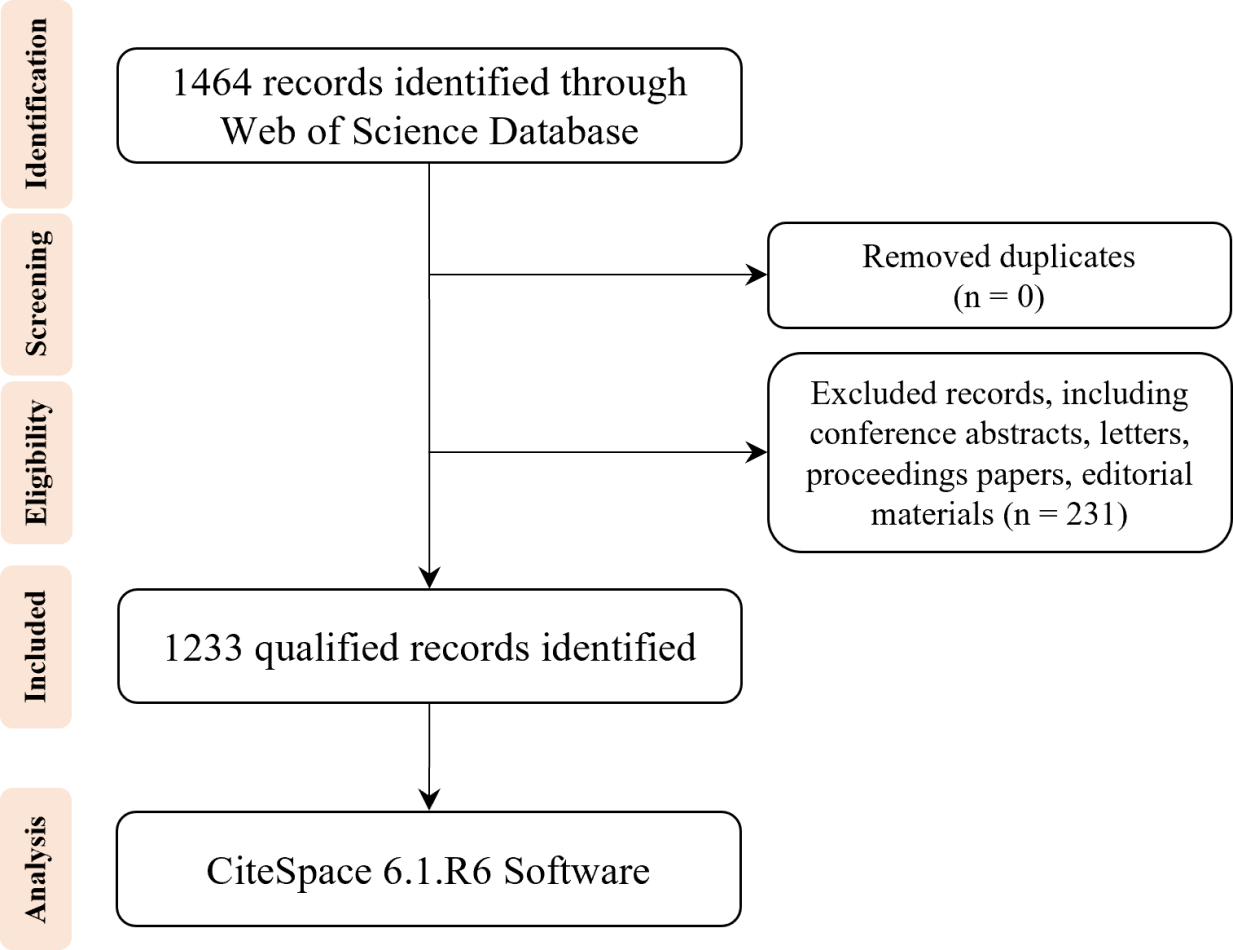
Study flowchart of the retrieval strategy.

All published data used in the study were acquired on the WOS platform from the SCI-EXPANDED, SSCI, CPCI-S, CPCI-SSH, A&HCI, ESCI, IC, and CCR-E databases. The search strategies were: Topic = (“DNA methylation” or “RNA methylation”) AND Topic = (“type 2 diabetes”). A total of 1,464 records were collected, and the search period has been validated until 31 December 2022. Subsequently, the duplicates were removed, and the following exclusion criteria were used: (1) conference abstracts; (2) letters to editors; (3) proceedings papers; (4) editorial materials. Finally, 1,233 records limited to articles and reviews were included for bibliometric analysis. The selection strategy is exhibited as a flowchart and illustrated in **Figure 1**.

### Data analysis

CiteSpace software (version 6.1.R 6), developed by Professor Chaomei Chen, was utilized to visualize the countries/regions, institutions, authors, and keywords (13, 14). The collaborative map presents a network as a node-link diagram. For nodes, the size is usually regarded as the frequency, the different colors correspond to different years, and the line between nodes represents co-occurrence or co-referencing relationships. The high centrality of nodes predicts the turning point of the research, which has a role to play in the network. The data analysis process has been performed in strict accordance with the guidance of the CiteSpace software and analyzed at least two times to ensure the accuracy of the output results. The analysis not only helps track key paths and milestones of research in the field but also provides a guiding perspective for future directions.

## Results

### Distribution and characteristics of annual publications

**Figure 2:**
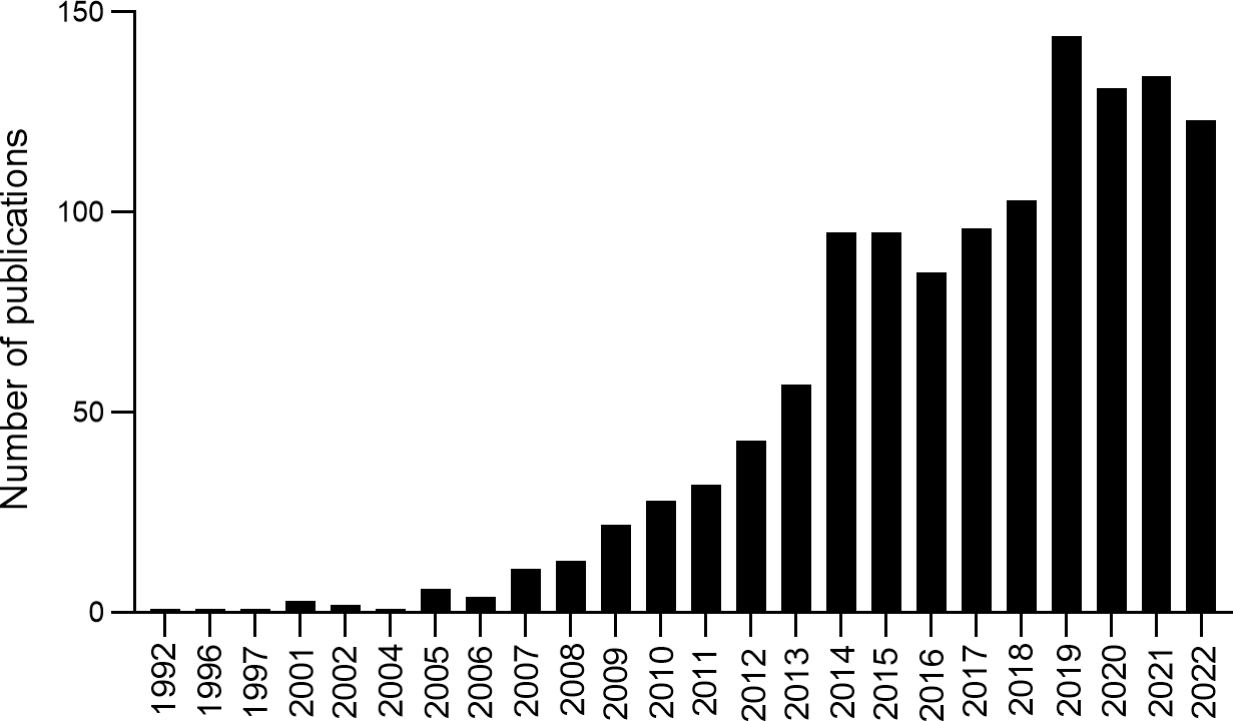
Annual number of publications and growth trends contributing to DNA/RNA methylation modification in T2DM from 1992 to 2022.

A total of 1,233 records were obtained from 1992 to 2022, and the number of publications per year was explored (**Figure 2**). The developmental course over the last 30 years exhibited three periods: initiation, continued development, and substantial growth. The first period (1992–2006) is the initiation of research, with fewer than five publications every year and the research growing slowly. The second period (2007–2013) represents continued development, with approximately 30 publications every year; outputs increased from 11 in 2007 to 57 in 2013. The third period (2014–2022) is the substantial growth phase, with increased publications from 95 in 2014 to 123 in 2022. The analysis indicated that more and more studies focused on the correlation between T2DM and DNA/RNA methylation modifications, suggesting that researchers are paying increasing attention to methylation modifications in T2DM.

### Analysis of scientific collaboration network

**Figure 3:**
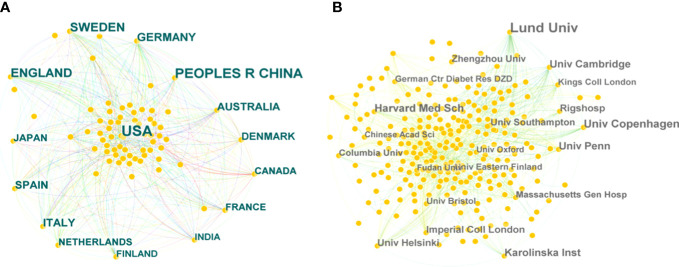
Bibliographic analysis of the scientific collaboration network. **(A)** Collaboration among countries/regions. **(B)** Collaboration among institutions. Different sizes denote the quantity of publications for countries or institutions, and the distance between the two circles represents the relatedness between them.

A collaborative network of countries was presented, with 81 nodes and 556 links (**Figure 3A**). This indicates that 81 countries have contributed to the field of T2DM and DNA/RNA methylation modification research. The top 10 countries ranked by publication counts, the earliest published year, and centrality are shown in detail in **Table 1**. The network showed that the main contributor was the USA, accounting for approximately 40% of publications (371), and it published earlier than any other country. The number of publications in China ranked second (241), followed by England (135), Sweden (120), and Germany (80). This comprehensive analysis implies that the USA is the most influential country in terms of studying T2DM and DNA/RNA methylation modifications.

**Table 1:** Top 10 countries in number of publications related to T2DM and DNA/RNA methylation modifications.

Rank	Publications	Countries	Years
1	371	USA	1992
2	241	China	2008
3	135	England	2007
4	120	Sweden	2007
5	80	Germany	2002
6	71	Italy	2007
7	63	Australia	2009
8	61	Spain	2010
9	56	Denmark	2007
10	45	Japan	1997

Further analysis illustrated a network of institutions and their cooperative relationships with 426 nodes and 1,273 links (**Figure 3B**). Of the 426 institutions, the top five contributing institutions in this field are Lund University, the University of Copenhagen, Harvard Medical School, the University of Cambridge, and the Karolinska Institute. Lund University was the most important node with the highest publication count (73 publications). In addition, the top five institutions in terms of centrality scores from publication were Lund University (0.12), the University of Pennsylvania (0.08), the University of Navarra (0.06), Columbia University (0.06), and the University of Cambridge (0.06). In terms of publication and centrality, Lund University was the core of this complex cooperative network.

### Analysis of publication authors and cited authors

**Figure 4:**
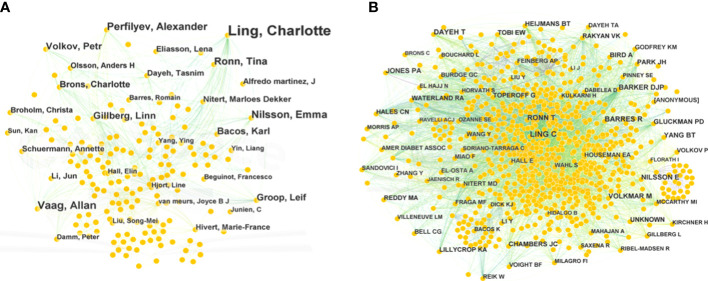
Bibliographic analysis of publication authors and cited authors. **(A)** Network of publication authors. **(B)** Network of cited authors. Different sizes denote the quantity of publications for authors or cited authors, and the distance between the two circles represents the relatedness between them.

The authors of 1,233 publications were displayed in a network that had 640 nodes and 1,726 links (**Figure 4A**). This network indicated that there were 640 authors, which comprised collaborative groups and scattered authors. The top five contributing authors were Charlotte Ling (53 publications), Allan Vaag (15), Emma Nilsson (16), Alexander Perfilyev (17), and Tina Ronn (14). Charlotte Ling and Tina Ronn collaborated on the epigenetic signatures of human tissues relevant to metabolism, potentially opening new ideas for T2DM therapy (7). Moreover, they verified that age was a possible factor affecting the DNA methylation of OXPHOS and participation in metabolic processes (17). This network implies that authors and groups are highly influential.

The network of cited authors had 974 nodes and 5,821 links (**Figure 4B**). Among the cited authors, Ling had the highest citation count (225), followed by Dayeh (162). In terms of centrality, the top five authors were Feinberg (0.18), Barker (0.11), Ahlgren (0.10), Barlow (0.09), and Gluckman (0.09) (**Table 2**). Ling is the author with the most published works and a high centrality, implying that he made marked contributions to research in the field of T2DM and methylation modifications. Ling worked at Lund University and predominantly focused on the relationship between DNA methylation and insulin target organs, including adipocytes, pancreatic islets, and skeletal muscle (16–18).

**Table 2:** Top 10 cited authors related to T2DM and DNA/RNA methylation modifications.

Rank	Frequency	Author	Rank	Centrality	Author
1	225	Ling	1	0.18	Feinberg
2	162	Dayeh	2	0.11	Barker
3	145	Barres	3	0.10	Ahlgren
4	141	Ronn	4	0.09	Barlow
5	126	Jones	5	0.09	Razin
6	122	Volkmar	6	0.09	Hales
7	120	Nilsson	7	0.09	Gluckman
8	120	Barker	8	0.08	Heijmans
9	110	Yang	9	0.08	Jones
10	107	Heijmans	10	0.07	Rakyan

### Analysis of publication journals

**Figure 5:**
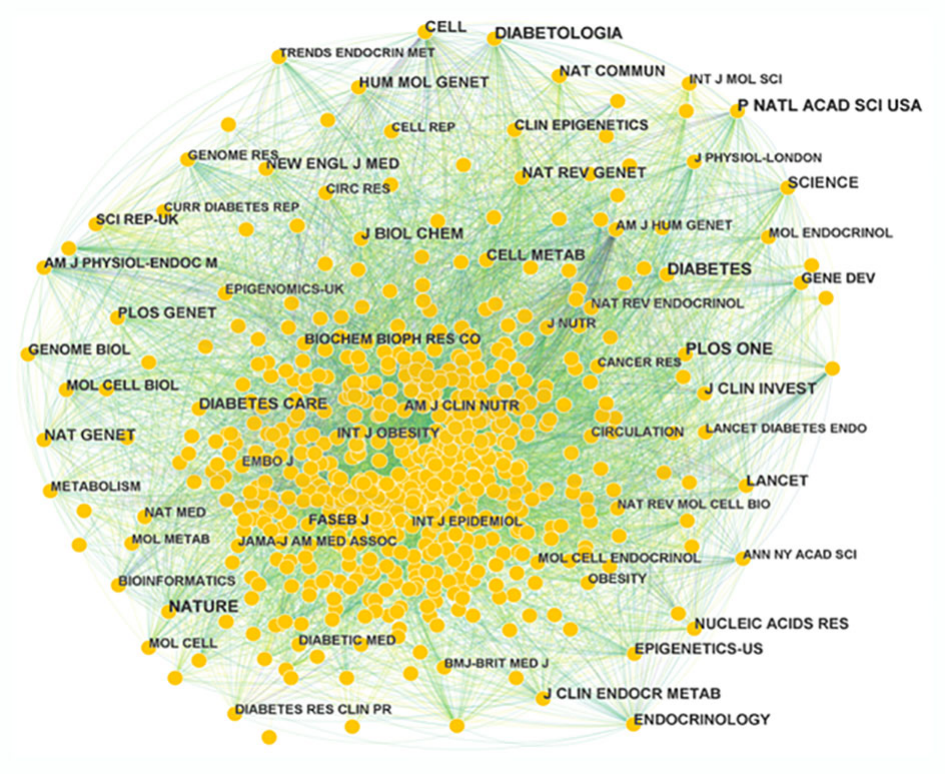
Map of publication journals. Different sizes denote the quantity of publications for publication journals, and the distance between the two circles represents the relatedness between them.

A total of 710 journals have contributed to the field of T2DM and DNA/RNA methylation modification research. A collaborative network of published journals was generated with 710 nodes and 7,476 links (**Figure 5**). The important nodes and links in the diagram may reveal the influential role of the publications on the research frontier. The journals with relatively high centrality were Am J Hum Genet (0.08) and Am J Clin Nutr (0.06). The top 10 cited journals related to T2DM and DNA/RNA methylation modifications are displayed in **Table 3**, and the top 10 journals related to epigenetics are presented in **Table S1**. In terms of citation counts, DIABETES had the highest citation counts (899), followed by PLOS One (785), and Nature (759). Among the cited journals, Nature, Cell, and Nat Genet had the highest Journal Impact Factors at 69.504, 66.85, and 41.307, respectively, indicating the research field of T2DM and DNA/RNA methylation modifications is a relative hotspot and novel.

**Table 3:** Top 10 cited journals that have published the most works related toT2DM and DNA/RNA methylation modifications.

Rank	Frequency	Cited Journal	Publication country	5-Year average IF^a^
1	899	DIABETES	USA	10.493
2	785	PLoS One	USA	4.069
3	759	Nature	ENGLAND	63.581
4	730	P Natl Acad Sci USA	USA	13.451
5	725	Diabetologia	GERMANY	10.617
6	589	J Biol Chem	USA	5.294
7	538	Nat Genet	USA	39.32
8	531	J Clin Invest	USA	19.232
9	526	Cell	USA	59.901
10	508	Diabetes Care	USA	17.243

^a^IF, impact factor according to Journal Citation Reports.

### Analysis of cited references

**Figure 6:**
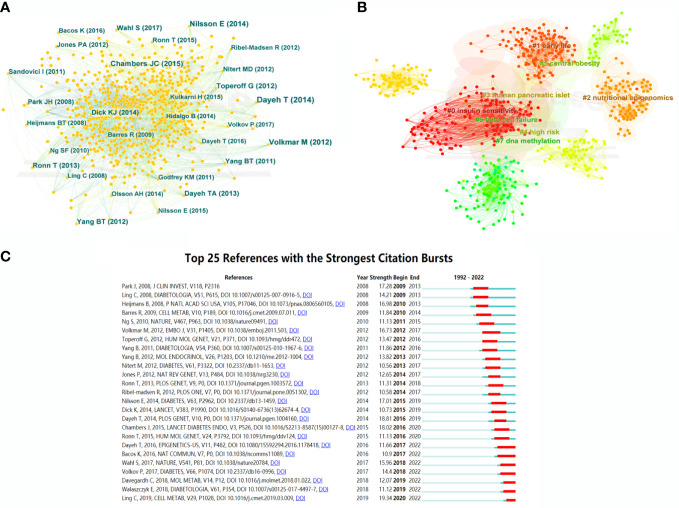
Bibliographic analysis of cited references. **(A)** Map of cited references. Different sizes denote the quantity of publications for cited references, and the distance between the two circles represents the relatedness between them. **(B)** A clustering map of topics for cited references. Each area with a colorful node represents the keyword clusters of the cited references. **(C)** The top 25 references with the strongest citation burst in T2DM-methylation modification research.

A co-citation network of cited references was further displayed, with 987 nodes and 4,223 links, which represented the relationship between research references and co-citations, respectively (**Figure 6A**). In terms of citation frequency, Dayeh had the highest citation count with an article published in 2014. This article revealed 479,927 methyl-CpG sites when comparing pancreatic islets from T2DM patients and non-diabetic donors, which provided novel targets for T2DM therapy (15). The second most-cited reference was that of Volkmar, published in 2012. This article unfolded a comprehensive DNA methylation profiling, implying the significant involvement of epigenetic dysregulation in human diabetic islets (19). The third most-cited study was published by Nilsson in 2014 and presented multiple transcriptional and epigenetic changes in adipose tissue related to the progression of T2DM based on whole genome expression and DNA methylation (20). In terms of centrality for cited references, the author with the highest ranking was Feil, who published an article in Nat Rev Genet and uncovered the relationship between epigenetics and the environment (21). Additionally, the epigenetic alterations in T2DM liver with reduced folate levels investigated by Nilsson in J Clin Endocrinol Metab ranked second, revealed the importance of epigenetic and transcriptional changes in the human diabetic liver, and suggested that the reduced folate contents might be an explanation for this (22).

Cluster analysis of the cited references was performed to investigate the distribution based on topic and time zone. This clustering was divided into eight clusters (Q-value = 0.7339, S-value = 0.854), with high credibility and an effective clustering. As shown in **Figure 6B**, the most-cited topics have mainly focused on insulin sensitivity, DNA methylation, nutritional epigenomics, obesity, human pancreatic islets, and early life. The cited references highlighted a greater interest in the research of epigenetic modifications and T2DM. **Figure 6C** shows the top 25 cited references with the strongest bursts from the earliest mention to December 2022. The first two cited references dissect the research field of T2DM and epigenetics, which has been underway since 2009 and has attracted continuous attention until now.

### Analysis of keywords

**Figure 7:**
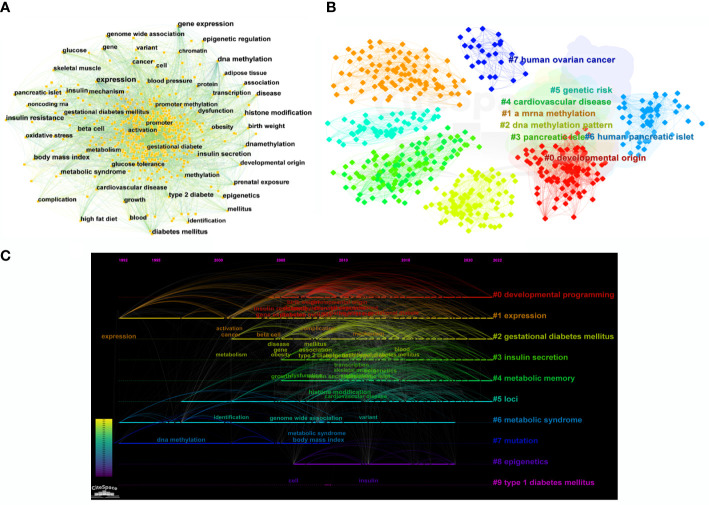
Bibliographic analysis of keywords. **(A)** Visualization of keywords. Each node denotes the keywords. **(B)** A clustering map of keywords. Colorful areas with the nodes represent the keyword clusters. **(C)** A timeline view of keywords. The horizontal line with different colored labels denotes the cluster formed by the keyword, and the position of the node on the line represents the year in which the publication containing the keyword first appeared.

According to the bibliometric theory of literature, the keywords reflect the research trends and hotspots in a field. The frequency of the words in a certain period indicates that an emerging topic has appeared in the research (23, 24). By using Citespace software, a collaborative network of keywords was produced from 1992 to 2022, with 569 nodes and 4,269 links (**Figure 7A**). In total, 541 research keywords were detected in the field of T2DM with methylation modifications, highlighting the hottest topics in this field. In terms of frequency and centrality (**Table 4**), the highest scores were for DNA methylation, gene expression, and diabetes, which indicates that current research pays more attention to the relationship between T2DM and epigenetics. The epigenetic events would contribute to and investigate the underlying mechanisms of the pathogenesis of diabetes (25).

**Table 4:** Top 10 keywords of the publications related to T2DM and DNA/RNA methylation modifications.

Rank	Keyword	Frequency	Rank	Keyword	Centrality
1	DNA methylation	577	1	expression	0.19
2	gene expression	231	2	DNA methylation	0.14
3	insulin resistance	216	3	diabetes mellitus	0.09
4	expression	215	4	gene expression	0.09
5	type 2 diabetes	164	5	molecular mechanism	0.07
6	adipose tissue	113	6	histone modification	0.07
7	obesity	106	7	body mass index	0.07
8	gene	100	8	insulin resistance	0.07
9	risk	95	9	beta cell	0.06
10	association	88	10	disease	0.06

The cluster analysis of the keywords was further illustrated (**Figure 7B**). Eight clusters were formed (Q-value = 0.3535, S-value = 0.6841), indicating that clustering was reasonable and the community structure was significant. The top five keywords were “developmental origin,” “RNA methylation,” “DNA methylation,” “genetic risk,” and “pancreatic islet.” Among these keywords, “DNA methylation” exhibited the highest frequency with 577 citation studies, demonstrating that it is a research hotspot in the field of T2DM. Methylation modification includes DNA methylation and RNA methylation. Based on recent trends, current research has begun to pay considerable attention to RNA methylation, suggesting that RNA methylation may have excellent research potential. From the timeline view (**Figure 7C**), it shows the evolution of epigenetic research in T2DM. In terms of color warmth, “epigenetics” has become part of the latest studies in the research of developmental programming, insulin secretion, metabolic memory, and syndrome in the field of T2DM. The variation of methylation modifications highlights the significant effect on the risk of diabetes and indicates that it may be a key factor in the pathogenesis of T2DM (26).

## Discussion

Methylation modification is a highly dynamic and reversible process that regulates gene expression. Increasing evidence has suggested that epigenetics, especially methylation modifications, could provide new strategies for understanding the pathogenesis of T2DM (27, 28). Targeting these epigenetic processes provides a promising opportunity to prevent and treat T2DM (7). In this study, a bibliometric analysis was conducted to provide a comprehensive overview of global hotspots and trends in T2DM and DNA/RNA methylation research over the past 30 years. The cluster analysis revealed that methylation has received increasing attention and is of great importance for guiding future trends in this research field.

With the recognition of the significance of methylation modifications in T2DM, an increasing trend in publications and citations on methylation in T2DM has been observed every year since 2014. This indicates that T2DM research has had a rapid development in the field of methylation, and especially the methylation modifications in T2DM constitute a hot new area. These publications include 1,127 research articles (693 involving human studies and 434 involving animal studies) and 106 reviews. Using Citespace software, a bibliographic coupling analysis was conducted to clarify the literature based on countries, institutions, authors, and keywords. Among the publications, the USA and Lund University are the most influential countries and institutions, respectively. Charlotte Ling made the most outstanding contribution to the field, and the most popular journal is DIABETES. The collaborative network of keywords identified three potential research trends in methylation and T2DM, including developmental origin, insulin resistance, and metabolism. This analysis clarified the relationship between previous and future research trends.

The visualization network is an effective method of tracking research progress (29). Of these 1,233 records, 1,179 publications are involved in DNA modification, and 54 publications are associated with RNA modification. The improvement of technological capabilities makes us have a deeper understanding of the role of DNA/RNA methylation modifications, such as regulatory elements, gene bodies, and transcriptional start sites. The technologies for DNA methylation are primarily involved in bisulfite conversion, endonuclease digestion, and affinity enrichment (30). Although the first studies published firt published in 1980 used quantitative reversed-phase high performance liquid chromatography, most recent publications conducted DNA methylation using high-throughput sequencing techniques (31, 32). DNA methylation is the addition of a methyl group attached to either the cytosine or adenine nucleotide of a DNA molecule, thereby affecting the expression of genes at the transcriptional level (8). According to our analysis, most studies consistently reported differential DNA methylation that affected PDX-1, GLP1R, PPARGC1A, etc., and thus led to insulin resistance (16, 18, 33). Moreover, some studies reported DNA methylation profiles in several genes related to RXRA, SREBF1, PPARA, etc., closely associated with age-related susceptibility to hepatic insulin resistance (34) and increased liver lipid metabolism (35). The first studies of DNA methylation alterations in humans can be traced back a dozen years and were performed on skeletal muscle and pancreatic islets from volunteers with T2DM (36, 37). Tissues of diabetic patients were compared to those of healthy individuals and altered patterns of DNA methylation were observed. According to the top 10 publications, most studies on DNA methylation and T2DM were done among Europeans, European ancestry Americans, Indian Asians, and Mexican Americans using adipose, liver, and whole blood tissues (20, 38, 39). Alterations in DNA methylation reported by these studies were related to differential gene expression, such as INS and PGC-1α, which may contribute to understanding the phenotypes characterized in T2DM.

Furthermore, an increasing interest in studies linking RNA methylation to T2DM has become important since 2019 due to technical and bioinformatic advances. RNA methylation is the most abundant and reversible modification that is controlled by methyltransferases and demethylases (40). The literature shows that RNA methylation could regulate mRNA expression encoded by genes such as FOXO1, G6PC, and FASN, which control hepatic lipid and glucose metabolism, insulin sensitivity, and human β-cell biology (41–43). Current studies have analyzed the RNA methylation of several candidate genes encoded by PDX1, FASN, and IRS1 for T2DM using human pancreatic islets, liver, and heart tissues (42–44). The study of RNA methylation in relation to T2DM is still a relatively young field of research, but it has received much attention and grown at a fast pace. Regardless, DNA/RNA methylation modifications in T2DM are still complex and worth investigating. Full elucidation of DNA/RNA methylation modifications and the associated pathways they affect would provide possible therapeutic targets for the treatment of diabetes; therefore, we speculate that this direction will become a hotspot in the studies of T2DM.

This study revealed the status and trends of methylation in T2DM by bibliometric and visual analysis; however, there are some limitations to be considered. Firstly, the data was only collected from the WOS database, which might exclude some research results from other databases. In addition, although all possible keywords were considered, we cannot guarantee that there are no missing publications related to the topic. More importantly, owing to the low citation frequency, some promising and high-quality publications may not receive attention. Therefore, there should be a focus on the latest publications for a more accurate prediction of the hotspots and frontiers in the research.

## Conclusion

Although increasing research highlights the significance of methylation modifications in T2DM, a more comprehensive analysis is still needed. This study utilized the CiteSpace software to evaluate new perspectives concerning potential collaborators and cooperative institutions, status, and frontiers, thereby providing the future research trends for exploring and developing the pathogenesis of T2DM. In recent years, the primary research concerning epigenetics and T2DM has involved DNA methylation, while future research may shift the emphasis to RNA methylation studies. Overall, the findings from this study provide valuable information for guiding future research directions in the field of DNA/RNA methylation modification in T2DM.

## Data availability statement

The original contributions presented in the study are included in the article/**Supplementary Material**. Further inquiries can be directed to the corresponding author.

## Author contributions

CC designed the research and revised the manuscript. CJ contributed to data acquisition, analysis, and drafted the manuscript. YH and SW contributed to literature search and interpretation of the results. All authors listed have made a substantial, direct, and intellectual contribution to the work and approved it for publication.
