# Supplementary material for: Emerging trends in DNA and RNA methylation modifications in type 2 diabetes mellitus: a bibliometric and visual analysis from 1992 to 2022
Source: Front Endocrinol (Lausanne). 2023 May 2;14:1145067. doi: 10.3389/fendo.2023.1145067 (PMC10187586; doi:10.3389/fendo.2023.1145067)
Supplement: Supplementary file 1 [file Table_1.docx]

**Table S1** Top 10 journals related to epigenetics.

| Rank | Journal | Publication country | 5-year average IF^a^ |
| --- | --- | --- | --- |
| 1 | NATURE REVIEWS GENETICS | ENGLAND | 59.158 |
| 2 | NATURE GENETICS | USA | 39.320 |
| 3 | TRENDS IN ECOLOGY&EVOLUTION | ENGLAND | 21.005 |
| 4 | GENOME BIOLOGY | ENGLAND | 20.366 |
| 5 | MOLECULAR BIOLOGY AND EVOLUTION | USA | 20.074 |
| 6 | ANNUAL REVIEW OF GENETICS | USA | 17.642 |
| 7 | GENOME MEDICINE | ENGLAND | 15.579 |
| 8 | TRENDS IN GENETICS | NETHERLANG | 14.068 |
| 9 | GENES & DEVELOPMENT | USA | 13.623 |
| 10 | GENOME RESEARCH | USA | 13.537 |

^a^IF, impact factor according to Journal Citation Reports.
